# TADF Dye-Loaded Nanoparticles for Fluorescence Live-Cell Imaging

**DOI:** 10.3389/fchem.2020.00404

**Published:** 2020-05-08

**Authors:** Carina I. C. Crucho, João Avó, Ana M. Diniz, Sandra N. Pinto, José Barbosa, Poppy O. Smith, Mário Nuno Berberan-Santos, Lars-Olof Pålsson, Fernando B. Dias

**Affiliations:** ^1^IBB-Institute for Bioengineering and Biosciences, Instituto Superior Técnico, Universidade de Lisboa, Lisbon, Portugal; ^2^Department of Chemistry, Durham University, Durham, United Kingdom; ^3^Department of Physics, Durham University, Durham, United Kingdom

**Keywords:** TADF, dye-loaded nanoparticles, luminescent probes, optical imaging, fluorescence imaging, fluorescence microscopy

## Abstract

Thermally activated delayed fluorescence (TADF) molecules offer nowadays a powerful tool in the development of novel organic light emitting diodes due to their capability of harvesting energy from non-emissive triplet states without using heavy-metal complexes. TADF emitters have very small energy difference between the singlet and triplet excited states, which makes thermally activated reverse intersystem crossing from the triplet states back to the singlet manifold viable. This mechanism generates a long-lived delayed fluorescence component which can be explored in the sensing of oxygen concentration, local temperature, or used in time-gated optical cell-imaging, to suppress interference from autofluorescence and scattering. Despite this strong potential, until recently the application of TADF outside lighting devices has been hindered due to the low biocompatibility, low aqueous solubility and poor performance in polar media shown by the vast majority of TADF emitters. To achieve TADF luminescence in biological media, careful selection or design of emitters is required. Unfortunately, most TADF molecules are not emissive in polar media, thus complexation with biomolecules or the formation of emissive aggregate states is required, in order to retain the delayed fluorescence that is characteristic of these compounds. Herein, we demonstrate a facile method with great generalization potential that maintains the photophysical properties of solvated dyes by combining luminescent molecules with polymeric nanoparticles. Using an established swelling procedure, two known TADF emitters are loaded onto polystyrene nanoparticles to prepare TADF emitting nanomaterials able to be used in live-cell imaging. The obtained particles were characterized by optical spectroscopy and exhibited the desired TADF emission in aqueous media, due to the polymeric matrix shielding the dye from solvent polarity effects. The prepared nanoparticles were incubated with live human cancer cells and showed very low cytotoxicity and good cellular uptake, thus making fluorescence microscopy imaging possible at low dye concentrations.

## Introduction

Thermally Activated Delayed Fluorescence (TADF) emitters attracted great attention from both academia and industry due to their potential for efficient triplet harvesting in organic light emitting diodes (OLEDs) (Yang et al., 2017). In OLEDs charge recombination results in the formation of singlets and triplet states in a ratio 1:3. As the triplet state is often non-emissive, the internal efficiency of OLEDs is limited to 25%. The need to improve device efficiency triggered the application of heavy-metal complexes in the OLEDs field (Baldo et al., 1998). However, metal-based phosphors may create potential problems in industries with high production rates. Moreover, these complexes show tendency to chemically degrade during the vacuum deposition process used in device fabrication, often show short working lifetimes in devices, and more important, there are no stable complexes with emission in the deep-blue region, which is essential for both high quality displays and lighting. TADF molecules promised to solve these problems in an elegant way (Uoyama et al., 2012).

TADF molecules create excited states with strong charge transfer character (CT) which can have vanishingly small electron exchange energies, resulting in nearly equivalent singlet and triplet energies. Using thermal energy to promote the up-conversion of non-emissive triplet states back to the singlet manifold is thus efficient through reverse intersystem crossing (RISC), thereby giving a method of “harvesting” up to 100% of triplet states formed by charge recombination in OLEDs (Dias et al., 2013). TADF emitters can thus combine the most desirable properties of metal-carrying phosphorescent emitters, namely 100% triplet harvesting, with the added benefit of the long-term stability of a fluorescent emitter. This is what both display and lighting manufacturers seek, but as yet not been materialized fully in the blue spectral region (Bui et al., 2018).

Due to the involvement of long-lived triplet states, the luminescent from TADF molecules shows two clear time regimes. A first component, appearing due to the decay of singlet states created immediately after excitation, shows lifetimes in the order of few nanoseconds. This component is often referred as the prompt fluorescence (PF) and shows small variation with temperature or the presence of oxygen. A second luminescent component, appearing due to the up conversion of triplet states to the emissive singlet manifold, usually shows lifetimes that range from a few microseconds to milliseconds and is identified as delayed fluorescence (DF). The DF intensity can show strong variation with temperature and the presence of oxygen (Baleizão et al., 2008; Dias et al., 2017). Besides lifetime, PF and DF also contrast their variation with temperature. While the PF tends to decrease in intensity with increasing temperature (due to the emergence of non-radiative pathways), the DF intensity does exactly the opposite. Essentially, this happens because the DF component appears from a process involving the reverse intersystem crossing between the triplet and singlet states, which are separated by a small energy difference. TADF is thus a thermally activated mechanism, which is strongly affected by intra-molecular phenomena that can influence the triplet state, e.g., internal conversion, singlet-triplet energy splitting, intersystem crossing, triplet quenching mechanisms, medium properties etc. The DF is, therefore, extremely sensitive to the presence of oxygen, temperature, the dielectric medium, and may even vary due to the presence of analytes. As an example, if the TADF molecule selectively interacts with an analyte, most probably this will have a significant effect on the properties of the triplet excited state of the TADF emitter. These changes will be inevitably reflected in the TADF efficiency and lifetime (Chen and Song, 2019).

The DF component can be used as a long-lived luminescence to enable time-gated acquisition methods that ignore nonspecific and non-negligible resolution-limiting signals from autofluorescence and light-scattering (Zhao et al., 2011). For these reasons, TADF emitters have attracted significant scientific interest as an alternative to heavy metal-based complexes in optical imaging (Chen and Song, 2019). Despite these very interesting properties the application of TADF molecules in the optical imaging and sensing remains largely unexplored. Since most TADF dyes are weakly soluble in water and show very weak luminescence in polar solvents due to their charge transfer properties, their direct use in biological media has been critically hindered. To overcome these drawbacks, TADF emitters have been encapsulated in macromolecules (Xiong et al., 2014), precipitated onto nanoaggregates (Ni et al., 2018, 2019; Zhu et al., 2018) or assembled in amphiphilic polymer micelles with other small-molecular-weight dopants (Li et al., 2017; Luo et al., 2019; Tsuchiya et al., 2019). In addition, we previously reported the preparation of TADF emitting silica nanoparticles using a simple doping procedure with a silane-bearing TADF derivative (Crucho et al., 2020). These approaches were successful in achieving TADF emission in aqueous media, and even in mitigating oxygen quenching, providing an interesting platform to develop TADF-based optical probes for time-resolved optical imaging. However, most reported strategies rely on particular structural features of TADF emitters to allow complexation with macromolecules, aggregated induced or enhanced emission or synthetically challenging dye derivatization. On the other hand, polymer micelle doping procedures can be applied to a wider range of TADF molecules but require demanding and small-scale preparation steps.

Herein we explore the viability of conventional TADF dyes encapsulated in a polymeric nanocarrier as luminescent probes for optical fluorescence microscopy studies in cells. Luminescent nanomaterials are increasingly gaining relevance for biological and biomedical applications, offering many advantages over conventional molecular probes (Wolfbeis, 2015; Yu et al., 2017; Peng et al., 2018). Amongst them, dye-doped fluorescent nanoparticles exhibit improved biocompatibility, photostability, and optical brightness when compared to free dyes, and find application in biological optical imaging, theragnostic, and chemical sensing (Montalti et al., 2014; Li et al., 2019). In this work, we describe the preparation of TADF emitting polymeric nanoparticles using a simple straightforward swelling procedure, which is advantageous for the encapsulation of dyes with strong potential for generalization. Two well-studied TADF emitters (Wang et al., 2014; Dias et al., 2016) were selected and encapsulated in polystyrene (PS) nanoparticles. PS is a versatile and attractive matrix for dye encapsulation, being commercially available as nanoparticles in a broad size range and has been extensively used in the encapsulation of organic and inorganic fluorophores for imaging and sensing applications (Mader and Wolfbeis, 2010; Lee et al., 2013). However, to the best of our knowledge, PS nanoparticles have never been used for the encapsulation of TADF emitters. In this respect, we studied the influence of dye encapsulation and aqueous dispersion on the optical properties of these TADF emitters and evaluated the applicability of their dye-loaded PS nanoparticles in live-cell fluorescence microscopy imaging.

## Results and Discussion

### Synthesis and Preparation of TADF Dye-Loaded Nanoparticles

**Scheme 1:**
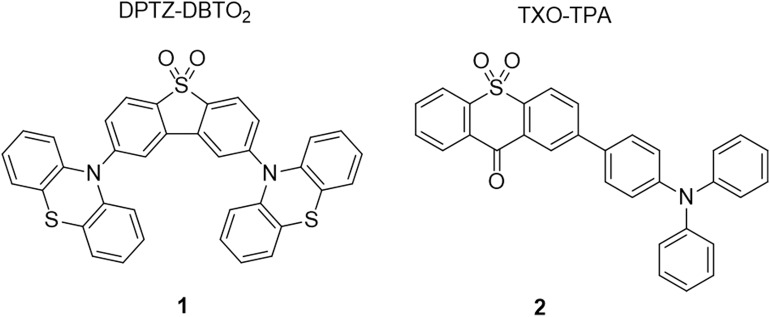
Chemical structures of TADF emitters used for the preparation of luminescent nanoparticles.

For the preparation of TADF emitting nanoparticles and to evaluate the effects of dye-encapsulation in the particle matrix, we selected two known TADF emitters (Scheme 1). Both compounds were previously studied toward their TADF properties and were successfully applied in the preparation of OLEDs with good to excellent external quantum efficiencies (Wang et al., 2014; Dias et al., 2016). 2,8-Di(phenothiazine-10-yl)dibenzothiophene-*S,S*-dioxide (DPTZ-DBTO_2_, dye **1**) is a well-known TADF emitter that displays highly efficient triplet harvesting in nonpolar media through the electronic coupling between the local triplet (^3^LE) and singlet and triplet charge transfer states (^1^CT and ^3^CT respectively). However, in polar solvents, the energy level of the charge transfer states are reduced, drastically affecting the TADF efficiency. This makes dye **1** a good candidate to probe the polarity of polymeric nanoparticles and the dye-encapsulation effect on the TADF properties. Synthesis of DPTZ-DBTO_2_, dye **1**, was prepared following published procedures (Dias et al., 2016). The dye was characterized by NMR (^1^H, ^13^C) and compound purity was established by elemental analysis. Commercially available 2-(4-triphenylamino)-thioxanthenone (TXO-TPA, dye **2**), was also selected in order to evaluate the generalization potential of the preparation method of TADF emitting nanoparticles. Similarly, to dye **1**, this dye displays a small singlet-triplet energy gap (ΔE_ST_) in nonpolar media and exhibits strong polarity dependence of its optical properties (Nobuyasu et al., 2019).

Aiming at a straightforward procedure for the preparation of TADF-emitting particles using non-functionalized fluorophores, we selected a well-established swelling procedure based on the adsorption of hydrophobic dyes onto the matrix of polymeric nanoparticles (Behnke et al., 2011). This method allows the preparation of dye-loaded nanomaterials using pre-formed polymeric nanoparticles, resulting in optical probes with enhanced stability, biocompatibility and applicability in biological media (Lee et al., 2011; Wang et al., 2013). For this purpose, polystyrene (PS) nanoparticles were chosen not only because nanosized and spherical particles are easy to synthesize, but also due to their commercial availability in a wide range of sizes and distinct surface characteristics. In addition, PS is permeable to oxygen, allowing the direct evaluation of the TADF properties of the luminescent nanomaterials in aqueous suspension. In this work, we used non-functionalized PS nanoparticles freshly prepared from emulsion polymerization procedures (PSP) and commercially available PS nanoparticles modified with positively charged amino-groups (PSNH_2_). This allowed us also to analyze the effect of nanoparticle surface functionalization on cell function and on dye loading and photophysics. While PSP can be easily obtained in large quantities using straightforward procedures, the lack of surface charge may hinder their application as optical probes in biological media.

**Figure 1:**
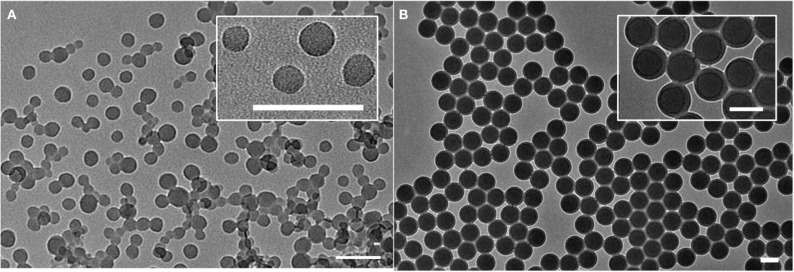
TEM images of the unloaded nanomaterials PSP **(A)** and PSNH_2_ **(B)**. Scale bar = 100 nm.

Non-functionalized PSPs were synthesized by emulsion polymerization. Following synthesis, the obtained suspensions were centrifuged. Due to the high hydrophobic character of these particles, stable aqueous suspensions are usually difficult to obtain from aggregated PSPs. For this reason and because aggregation could reduce cellular uptake, the centrifuged pellets were discarded and the supernatants were extensively dialyzed against water to remove surfactant molecules and traces of unreacted styrene monomer, which are the main source of PSPs cytotoxicity. The washed suspensions were characterized by TEM and exhibited nanoparticles of spherical morphology with diameters of 32 ± 4 nm, as shown in Figure 1A. The amino-modified particles (PSNH_2_) were also imaged by TEM, showing spherical morphologies with diameters of ca. 100 nm (Figure 1B).

Both dyes, **1** and **2**, were loaded into PSP and PSNH_2_ materials through swelling methods using tetrahydrofuran (THF) as organic solvent, and the effects of dye-loading on the physicochemical properties of obtained nanomaterials were studied. From TEM images and dynamic light scattering spectroscopy measurements, it is evidenced that the addition of dye does not significantly affect particle morphology and suspension stability (Figures S2–S4). The effects of the swelling step on size distribution and on the surface charge were also analyzed and compared to the amount of incorporated dye (Table 1). For dye **1**, it is shown that the incorporation in pristine polystyrene particles is more efficient when compared to the amine-modified polymer, with neutral particles (**PS1**) bearing over 3-fold more amount of dye than their charged counterparts (**PS2**) after isolation. Since the dye loading procedure is based on the hydrophobic interactions between dye and matrix, this difference can be attributed to the positively charged surface of PSNH_2_ nanoparticles that may affect dye adsorption. This effect was also noticed in the incorporation of dye **2**, with the loading into pristine polystyrene particles (**PS3**) being more effective than into the amino-modified material (**PS4**, Table 1). The different degree of dye-loading is also reflected on the increase in size of the nanomaterials after the swelling procedure. While the neutral PS nanoparticles undergo an increase of ca. 15%, this effect is less evident in the amino-modified counterparts (ca. 4%).

**Table 1:** Morphological properties of prepared and dye-loaded PS nanoparticles.

	**PSP**	**PSNH_**2**_**	**Dye 1@PSP (PS1)**	**Dye 1@PSNH_**2**_ (PS2)**	**Dye 2@PSP (PS3)**	**Dye 2@PSNH_**2**_ (PS4)**
Diameter^a^ (nm)	31.7	103.0	35.6	106.4	37.1	107.2
PDI	0.02	<0.01	0.02	0.01	0.02	0.01
Dye concentration (w/w %)	no dye	no dye	0.97	0.31	0.59	0.34

a *determined by TEM*.

### Photophysical Characterization of TADF-Dyes Encapsulated in PSP Nanoparticles

Following the preparation of the luminescent TADF nanomaterials, their optical properties were characterized and compared to those of free dyes. The aim is to evaluate the effects of encapsulation on the dyes photophysics, with a particular focus on their TADF properties. Table 2 summarizes the determined photophysical parameters regarding the emission wavelength (λ_em_), prompt fluorescence quantum yield (Φ_PF_) and lifetime (τ_PF_) and delayed fluorescence quantum yield (Φ_DF_) and lifetime (τ_DF_).

**Table 2:** Prompt fluorescence and total quantum yield (Φ_PF_, Φ_PL_), prompt and delayed emission lifetime (τ_PF_, τ_DF_) and wavelength (λ_em_) of dyes **1** and **2**, and luminescent nanomaterials **PS1-4**, measured in aqueous media.

	**λ_**em**_ (nm)**	**$\Phi_{\text{PF}}^{\text{a}}$**	**τ_**PF**_ (ns)**	**$\Phi_{\text{PL}}^{\text{b}}$**	**DF/PF ratio**	**τ_**DF**_ (μs)**
Dye **1**	563	0.02	6.59	0.02	n.d.^c^	n.d.^c^
**PS1**	550	0.05	4.58	0.18	2.6	5.11
**PS2**	556	0.03	4.60	0.11	2.7	2.89
Dye **2**	629	<0.01	6.3	<0.01	n.d.^c^	n.d.^c^
**PS3**	539	0.18	16.5	0.24	0.3	11.78
**PS4**	555	0.07	7.5	0.09	0.3	9.56

^a^*measured in aerated conditions*;

^b^*measured in degassed conditions*;

c *not detected*.

**Figure 2:**
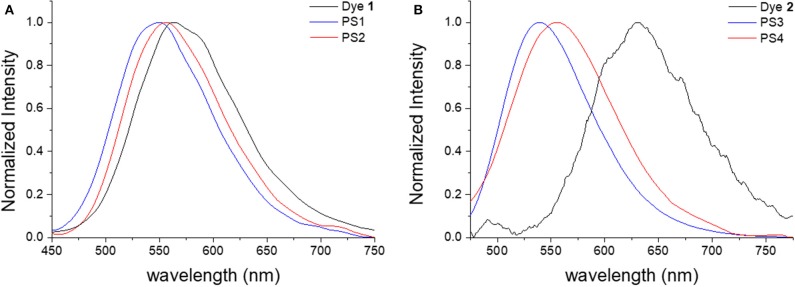
Normalized steady-state emission spectra of free dyes (black) and dye-loaded neutral (blue) and charged (red) nanoparticles in aqueous suspension: **(A)** dye **1**, **PS1**, and **PS2**, λ_ex_ = 340 nm; **(B)** dye **2**, **PS3**, and **PS4**, λ_ex_ = 420 nm.

Comparing the emission spectra of the prepared materials with those of free dyes in aqueous media, it is evidenced that the encapsulation of the dye leads to a blue-shift in the emission (Figure 2). While for dye **1** this effect is not significantly pronounced (e.g., $\lambda_{\text{em}}^{1}=$563 nm and $\lambda_{\text{em}}^{\text{PS}1}=$550 nm, Figure 2A), for dye **2** the emission is markedly shifted when the dye is incorporated in the nanomaterial (e.g., $\lambda_{\text{em}}^{2}=$629 nm and $\lambda_{\text{em}}^{\text{PS}3}=$539 nm, Figure 2B). The emission spectra of the dye-loaded nanomaterials is also significantly blue-shifted when compared to the emission of the dyes in most organic solvents (Figure S5). These results demonstrate the relatively low polarity of polystyrene matrix and the absence of water in the local environment of the dye, pointing to complete encapsulation. The difference in polymer polarity due to the modification with amine moieties can also be detected in the emission spectra of the nanomaterials. As observed in Figure 2, the emission of the neutral nanoparticles (**PS1** and **PS3**) is blue-shifted compared to that of their positively charged counterparts (**PS2** and **PS4**). These differences are more pronounced for the materials loaded with dye **2** ($\lambda_{\text{em}}^{\text{PS}3}=$539 nm vs. $\lambda_{\text{em}}^{\text{PS}4}$ = 555 nm) than those with dye **1** ($\lambda_{\text{em}}^{\text{PS}1}=$550 nm vs. $\lambda_{\text{em}}^{\text{PS}2}$ = 556 nm), due to the different polarity sensitivity of both dyes, in terms of optical properties.

**Figure 3:**
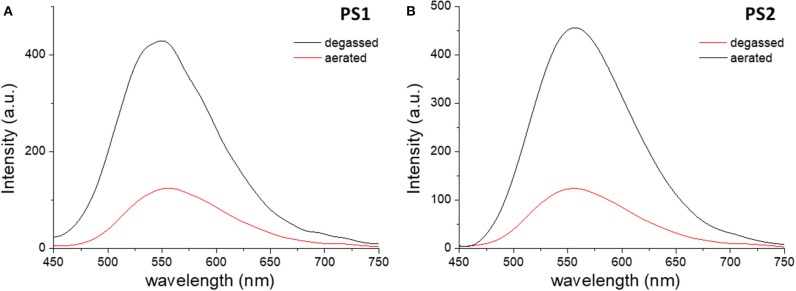
Steady-state emission spectra of **PS1 (A)** and **PS2 (B)** in aqueous suspension collected before (red) and after (black) degassing. λ_ex_ = 340 nm.

The effects of encapsulation on the luminescence quantum yields are also evident. Comparing the prompt fluorescence quantum yields of dye **1** in solution with those of materials **PS1** and **PS2** in aqueous suspension, it is evidenced the encapsulation into polystyrene nanoparticles enhances the prompt emission quantum yield (Φ_PF_) in fluid media, taking it to values closer to those of **1** in solid film (Table S1). Regarding the desired long-lived fluorescence, while degassed aqueous suspensions of dye **1** do not show any emission, **PS1** and **PS2**, suspended in water, exhibit an increase in the emission spectra intensity upon degassing (Figure 3).

**Figure 4:**
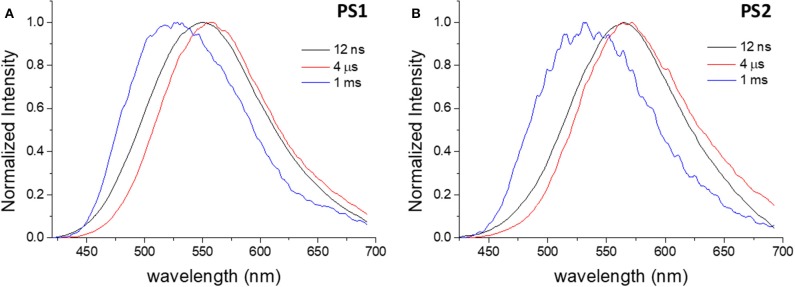
Normalized time-resolved emission spectra of **PS1 (A)** and **PS2 (B)** in degassed aqueous suspension, collected at different delay times. λ_ex_ = 355 nm.

Through time-resolved spectroscopy it is possible to verify that both materials show TADF and phosphorescence under degassed conditions (Figure 4). As previously described, in non-polar organic solvents and in solid film, dye **1** exhibits TADF ($\lambda_{\text{em}}^{\text{TADF}}$ = 560 nm) in the microsecond time-range, arising from reverse intersystem crossing that involves the local triplet excited state (^3^LE), the triplet (^3^CT)and the singlet (^1^CT) states of charge transfer character. As in previous studies of dye **1**, the phosphorescence is observed at long time delays (1 ms), and appears blue-shifted, when compared with the PF and DF spectra, peaking at $\lambda_{\text{em}}^{\text{Phos}}\;=$ 525 nm. Phosphorescence is dominated by the direct decay of the ^3^LE state to the ground state, which for dye **1**, in moderately polar solvents is slight above the CT manifold. Thus, it is demonstrated that the encapsulation of dye **1** in PS particles effectively preserves and transfers the optical properties of the free dye into the luminescent nanomaterials and enables TADF in aqueous media. Regarding the quantum efficiency of the long-lived emission and the DF/PF ratio, the determined values are substantially lower in PS particles (Table 2) than the reported values for nonpolar (zeonex) films (Dias et al., 2016; Nobuyasu et al., 2019). However, these results are consistent with the values obtained in more polar media, such as PS film and toluene (Table S1). Accordingly, the photoluminescence quantum yield (PLQY) is significantly higher for **PS1** than for **PS2**, and can be explained by polarity effects.

**Figure 5:**
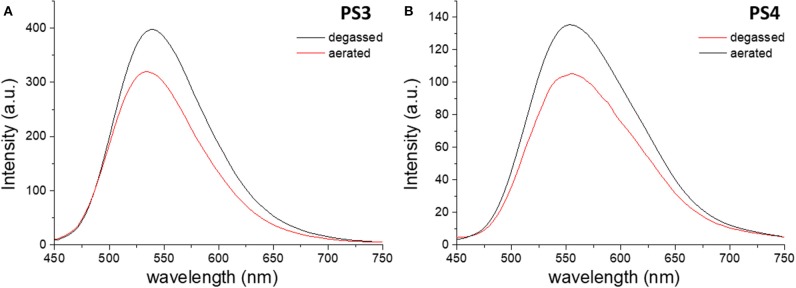
Steady-state emission spectra of **PS3 (A)** and **PS4 (B)** in aqueous suspension collected before (red) and after (black) degassing. λ_ex_ = 420 nm.

**Figure 6:**
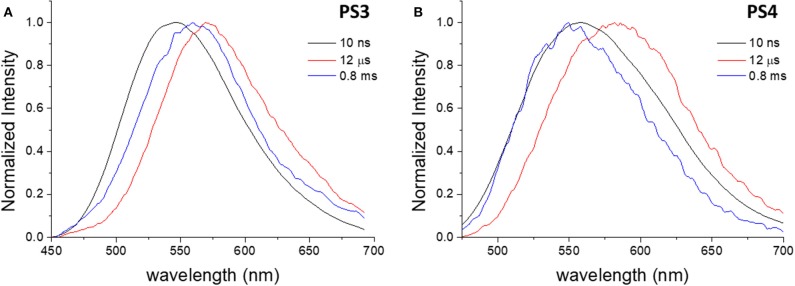
Normalized time-resolved emission spectra of **PS3 (A)** and **PS4 (B)** in degassed aqueous suspension, collected at different delay times. λ_ex_ = 355 nm.

In the case of dye **2**, the differences between free dye and dye-loaded materials are more evident. Comparing the emission in aqueous media, the nanomaterials show remarkably higher efficiency than the free dye, whose luminescence in water is almost negligible, and show an increase in emission intensity upon removal of oxygen (Figure 5). However, the increase in intensity due to oxygen removal is less than that observed for dye **1**. Figure 6 depicts the time-resolved emission spectra of **PS3** and **PS4**, suspended in water, where it is evidenced that a delayed fluorescence component is observed in the microsecond time range ($\lambda_{\text{em}}^{\text{TADF}}$ = 571 nm for **PS3** and $\lambda_{\text{em}}^{\text{TADF}}\;=$ 581 nm for **PS4**). Similarly to what is observed for materials loaded with dye **1**, both materials also exhibit a blue-shifted phosphorescence in the millisecond time-scale ($\lambda_{\text{em}}^{\text{Phos}}\;=$ 559 nm for **PS3** and $\lambda_{\text{em}}^{\text{Phos}}\;=$ 553 nm for **PS4**). Probably due to polarity effects, the PLQY value is ca. 3 times higher for **PS3** than for **PS4**, affecting both Φ_PF_ and Φ_DF_ values. Nevertheless, the DF/PF ratio is the same for both materials, suggesting that the reverse intersystem crossing step is not significantly affected. These results demonstrate that similarly to what occurred with dye **1**, the encapsulation of dye **2** in PS nanoparticles enables TADF detection in aqueous media.

**Figure 7:**
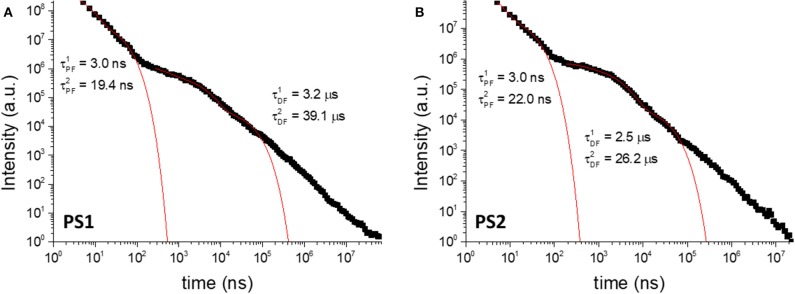
Luminescence decays of **PS1 (A)** and **PS2 (B)** collected in degassed aqueous suspension at room-temperature. λ_ex_ = 355 nm.

**Figure 8:**
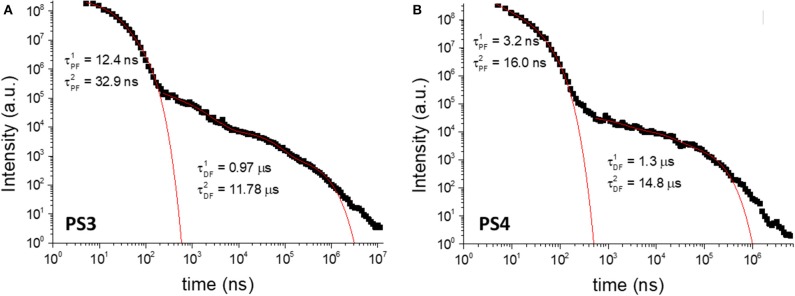
Luminescence decays of **PS3 (A)** and **PS4 (B)** collected in degassed aqueous suspension at room-temperature. λ_ex_ = 355 nm.

To further elucidate on the mechanism behind the long-lived emission, the full luminescence decay was measured for **PS1**/**PS2** (Figure 7) and **PS3**/**PS4** (Figure 8) at RT, and for **PS3** as a function of temperature (Figure S7A). Clear distinct luminescence decay regions are observed for dye **1** and dye **2** encapsulated in their respective nanoparticles. For dye **1** the PF decay is similar comparing **PS1** and **PS2**, but the DF decay becomes slightly faster in **PS2**. Interestingly, for dye **2** the DF decay is practically not affected from **PS3** to **PS4**, however the PF decay is significantly faster in **PS4**. These variations are probably mostly due to the effects of the medium on the compound ISC and RISC rates. For example, it is clear from the decays in Figures 7, 8 that the RISC rate is faster in **PS1**/**PS2** than in **PS3**/**PS4**. Comparing the intensity amplitudes at initial times for the PF and DF decays, **PS1**/**PS2** give DF/PF amplitude ratios ~10^−2^, whereas **PS3**/**PS4** give amplitude ratios around ~10^−3^ and ~10^−4^, respectively. Thus, the DF regime starts later in **PS3**/**PS4** than in **PS1**/**PS2**. This could be due to a slower RISC rate, but as the DF lifetime and the singlet-triplet energy gap are both similar to those of dye **1**, we have no evidence for this. Instead the stronger PLQY of the PF component indicates that the ISC rate is slower in **PS3**/**PS4**. Thus, fewer triplets are formed in these systems, compared to **PS1**/**PS2**, and thus weaker DF is observed. Nonetheless, the decays obtained as a function of temperature demonstrate that the long-lived component decaying in the μs range is associated with TADF, showing increasing intensity with temperature, as expected from a thermally activated process. Furthermore, the luminescence intensity measured as a function of excitation power shows a strictly linear variation, strongly suggesting that it is due to a unimolecular process (Figure S7B), compatible with TADF. The longer-lived emission decaying in the ms range, shows also a behavior that is compatible with phosphorescence emission. In contrast with the TADF decay, the emission associated with a decay time in the ms range increases at lower temperature. This indicates that the longer-lived emission is stronger as temperature decreases, which is consistent with phosphorescence.

In summary, these results clearly show that the encapsulation of hydrophobic TADF dyes in polymeric nanoparticles can be efficiently achieved using swelling methodologies and that this strategy results in an enhancement of their luminescent properties due to the shielding of solvent effects, allowing the application of these dyes in aqueous media.

### Live-Cell Imaging Tests With TADF Dye-Loaded Nanoparticles

To assess the applicability of the prepared nanomaterials in biological imaging, uptake, cytotoxicity, retention, and cell internalization of TADF functionalized materials were evaluated for immortalized human breast cancer (MCF-7) cells. Due to the fact that dye **1** does not absorb above 400 nm, it was not yet possible to test materials **PS1** and **PS2** in imaging scenarios, since the available excitation source for our confocal microscopy apparatus cannot excite in the UV range. For this reason, only materials **PS3** and **PS4** were tested.

**Figure 9:**
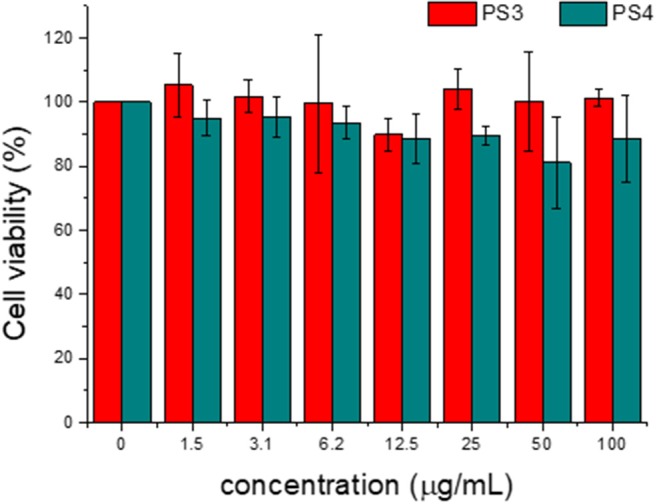
Effect of PS3 and PS4 on MCF-7 cell viability as a function of concentration in incubation medium for 24 h. The percentages refer to cell viability represented as a percentage of control.

Figure 9 depicts the cytotoxicity evaluation for these nanomaterials, assessed by measuring the cell metabolic activity with a colorimetric assay (results for dye **2** in Supplementary Material, Figure S8). It is evidenced that both pristine and modified PS particle display low dark cytotoxicity, with **PS3** virtually not affecting cell viability in the studied concentration range. The amino-modified **PS4** exhibits higher cytotoxicity, which can be attributed both to a positively charged surface and a larger size of these nanoparticles. This difference in cytotoxicity between neutral and positively charged particles is in agreement with previous reports (Saei et al., 2017).

**Figure 10:**
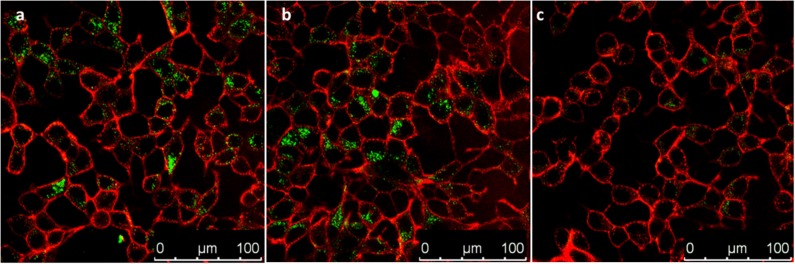
Confocal microscopy images of MCF-7 cells incubated for 24 h in the presence of **PS3** at different concentrations in incubation medium: **(a)** 100 μg/mL; **(b)** 50 μg/mL; **(c)** 25 μg/mL. Images show **PS3** emission in green and plasma membrane labeled with WGA-Alexa Fluor 633 in red.

**Figure 11:**
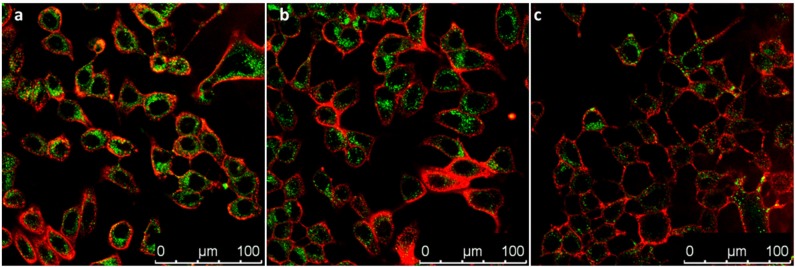
Confocal microscopy images of MCF-7 cells incubated for 24 h in the presence of **PS4** at different concentrations in incubation medium: **(a)** 100 μg/mL; **(b)** 50 μg/mL; **(c)** 25 μg/mL. Images show **PS4** emission in green and plasma membrane labeled with WGA-Alexa Fluor 633 in red.

**Figure 12:**
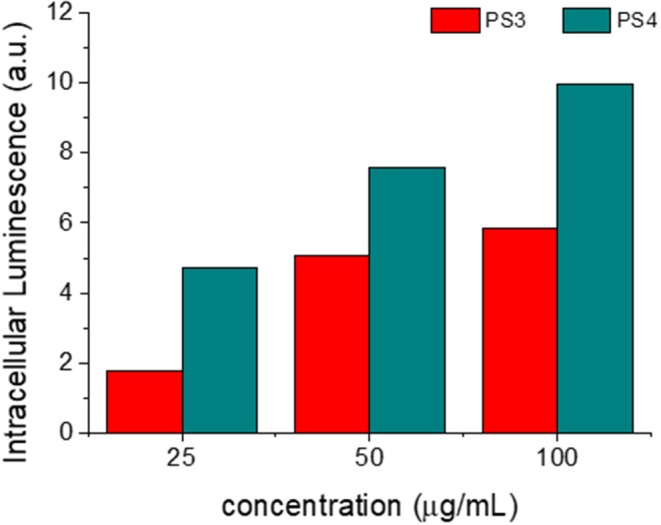
Integrated intracellular nanoparticle fluorescence, measured as a function of concentration of **PS3** (red) and **PS4** (blue) in the incubation medium. Analyzed area = 240 × 240 μm.

After this assessment, nanomaterials **PS3** and **PS4** were added to the incubation medium of MCF-7 cells for 24 h at different concentrations. Following this period, the cell membranes were stained with a selective dye and the resulting confocal microscopy images are depicted in Figures 10, 11. It is evident that both nanomaterials are internalized with a 24 h incubation period and that both materials are located primarily in the cytosol. However, comparing the images obtained for **PS3** with those for **PS4** (Figures 10, 11 and Figures S9–S11), two main differences become apparent. Firstly, the positively charged nanoparticles are more uniformly dispersed, with most of the cytosol exhibiting fluorescence from the nanomaterial at all tested concentrations. Conversely, **PS3** appears predominantly in the form of aggregates, which can be attributed to their neutral and hydrophobic surface. Secondly, the internalization and retention of **PS4** is significantly more efficient than for the neutral nanomaterial. Although the fluorescence quantum yield and dye concentration are lower in **PS4** than in **PS3**, the measured intracellular luminescence intensity is considerably higher for cells incubated with the positively charged particles (Figure 12 and Figure S12).

## Conclusions

The results shown in this work demonstrate that PS nanoparticles are efficient carriers for hydrophobic TADF emitters, enabling straightforward loading methodologies without requiring previous functionalization of luminescent dyes. Through an established swelling procedure, we prepared four luminescent nanomaterials, starting from two TADF emitters and two types of PS nanoparticles. The dye loading proved more efficient in pristine polystyrene nanomaterial than in the amino-modified analog. The encapsulation in polystyrene nanoparticles enhanced the optical properties of the TADF emitters in fluid media and even enabled the detection of delayed fluorescence and room-temperature phosphorescence in water. Due to the CT character of the excited states involved in the TADF process, the increase in polarity due to the presence of amine moieties led to an overall decrease in the PLQY of the positively charged nanoparticles. When added to the incubation medium of breast cancer cells, the dye-loaded nanoparticles exhibited very low dark cytotoxicity. Confocal microscopy revealed that both neutral and positive nanoparticles were internalized, locating primarily in the cytosol. The amino-modified particles were internalized more efficiently and were more uniformly dispersed inside the cells than the pristine polystyrene particles. We expect that this strategy can be generalized to a large variety of TADF dyes and extend their application beyond lighting devices. In order to achieve optimal DF/PF ratios for fluorescence imaging, care should be taken in the selection of TADF emitters, mainly regarding the polarity effects on the TADF process.

## Experimental

### Materials and Reagents

All chemical reagents were purchased from commercial suppliers without further purification unless otherwise noted. Styrene (St), Potassium persulfate (KPS), Divinylbenzene (DVB), Sodium dodecyl sulfate (SDS) were supplied by Sigma-Aldrich. KPS was purified via re-crystallization in deionized water. St and DVB were purified by passing through a basic alumina column. Amino-modified polystyrene nanoparticles (PSNH_2_) from Polybead® (type amino 100 nm spheres, 2.6 % suspension of solid particles in water) were purchased from Poly-sciences, Inc. (www.polysciences.com). Dialysis membranes with a molecular weight cutoff between 100–500 Da and a volume per length ratio of 1.8 mL cm^−1^ were obtained from Spectra/Por® Dialysis Membrane Biotech CE Tubing. Prior to usage, the membranes were soaked in distilled H_2_O for ~15 min and rinsed thoroughly with distilled H_2_O. All chemicals employed for synthesis were of reagent grade from TCI and used as received. For optical measurements, solvents were of spectroscopic grade and used without prior purification. Toluene was PA grade and was dried using metallic sodium and benzophenone and distilled before use. Tetrahydrofuran (THF) was of UV-spectroscopic grade and purchased from Sigma-Aldrich. Deionized water purified using a Millipore Milli-Q system to a resistivity of 18.2 MΩ was used throughout the experiments unless otherwise stated. Argon (Ar) gas (Alphagaz 1, 99,999%) was purchased from Air Liquid. Reagents and solvents used in synthesis were all analytical grade.

### Dye and Particle Synthesis/Preparation

#### Synthesis of DPTZ-DBTO_2_ (1)

The synthesis of **1** was carried out according to a previously reported strategy (Dias et al., 2016).

2,8-Dibromodibenzothiophene-S,S-dioxide (150 mg, 0.401 mmol, 1 eq.), phenothiazine (159 mg, 0.802 mmol, 2 eq.), were dried under vacuum for 0.5 h in a two-neck 25 mL round-bottomed flask fitted with a reflux condenser. The flask was back-filled with argon and dry toluene (9 mL) was added. The reaction mixture was bubbled with argon for 0.5 h, then Pd_2_(dba)_3_ (18.4 mg, 0.020 mmol, 5 mol%) and HP^t^Bu_3_BF_4_ (11.6 mg, 40.1 μmol, 10 mol%) were added and the reaction mixture was bubbled with argon for a further 0.5 h. NaO^t^Bu (11.6 mg, 0.12 mmol, 3 eq.) was added under a high flow of argon and the reaction was then stirred at 115°C for 21 h. Upon cooling, the solvent was removed at low pressure. The resulting residue was chromatographed (silica gel, eluent hexane/DCM 1:2 v/v) to give DPTZ-DBTO_2_ as a yellow solid (92.7 mg, 37.8%). Recrystallization of the residue from boiling dichloromethane followed by hot filtration and cooling at room temperature gave pale yellow crystals. ^**1**^**H-NMR** (400 MHz, DMSO-d6; Figure S1): δ = 7.99 (d, 2H, H_8_, ^3^*J*_H8−H7_ = 11.3 Hz), 7.69 (s, 2H, H_6_), 7.40 (d, 4H, H_4_, ^3^*J*_H4−H3_ = 9.9 Hz), 7.33 (d, 2H, H_7_, ^3^*J*_H7−H8_ = 11.1 Hz), 7.25 (t, 4H, H_2_, ^3^*J*_H2−H3, H2−H1_ = 10.1 Hz), 7.17 (t, 4H, H_3_, ^3^*J*_H3−H4_ = 9.9 Hz, ^3^*J*_H3−H2_ = 9.6 Hz), 7.01 (d, 4H, H_1_, ^3^*J*_H1−H2_ = 10.4 Hz). ^**13**^**C-NMR** (100 MHz, DMSO-d6): δ = 148.9, 141.8, 133.4, 133.1, 128.5 (C_4_), 128.3 (C_2_), 127.8, 125.8 (C_3_), 124.5 (C_8_), 123.0 (C_1_), 118.2, 115.4 (C_6_). **EA**. Calc. for C_36_H_22_N_2_S_3_·0.6 CH_2_Cl_2_, C 66.43; H 3.53; N 4.23. Found: C 66.35; H 3.45; N 4.11.

#### Synthesis of Polystyrene Nanoparticles (PSP)

PS nanoparticles were synthesized by an emulsion polymerization process (Zhang et al., 2015). A mixture composed of 0.25 g of SDS and 15 mL of water was first placed into a two-neck round-bottom flask equipped with a magnetic stirrer, a reflux condenser and an argon inlet. Purified styrene (1.68 mL) and DVB (0.25 mL) were added and the mixture was stirred under constant agitation (700 rpm) and purged with argon for 0.5 h. The temperature of the system was then raised to 70°C. 0.01 g of KPS was added with argon purging. After that, the reaction was allowed to occur for 5 h and argon was fluxed during the entire polymerization procedure. The mixture was cooled down to room temperature and sonicated for 15 min. The obtained PS nanoparticles were first centrifuged (30,000 rpm, 22°C, 3h). The supernatants were purified by repeated dialyses against distilled water (cellulose membrane, molecular weight cut-off 100–500 Da), obtaining a 1.0 % (w/v) suspension of solid particles in water. This stock suspension was stored at 4°C.

#### Dye-Loading Swelling Procedure

The swelling method has been described previously (Behnke et al., 2011). In a typical swelling procedure, dye (**1** or **2**) was dissolved in THF to give a stock solution. Dye loading of the PSP was performed by addition of the dye solution to an aqueous suspension of the PSP. This was then stirred at room temperature for 0.5 h. The solvent was allowed to evaporate overnight in a fume cupboard. The nanoparticle concentration in the remaining aqueous suspension was determined by drying and weighing a known volume.

##### Swelling procedure PS1

Dye **1** was dissolved in THF to give a stock solution (1.6 × 10^−3^ mol L^−1^). Dye loading of the PSP was performed by addition of 1.7 mL of the dye solution to 10 mL of an aqueous suspension of the PSP (1.0% w/v). After evaporation of the THF the suspension was centrifuged (2,000 g) and only the supernatant was used for the measurements.

##### Swelling procedure PS2

Dye **1** was dissolved in THF to give a stock solution (2.2 × 10^−3^ mol L^−1^). Dye loading of the PS-NH_2_ was performed by addition of 200 μL of the dye solution to 5 mL of an aqueous suspension of the PS-NH_2_ (0.5 % w/v).

##### Swelling procedure PS3

Dye **2** was dissolved in THF to give a stock solution (2.1 × 10^−3^ mol L^−1^). Dye loading of the PSP was performed by addition of 1.7 mL of the dye solution to 10 mL of an aqueous suspension of the PSP (1.0 % w/v). After evaporation of the THF the suspension was centrifuged (2,000 g) and only the supernatant was used for the measurements.

##### Swelling procedure PS4

Dye **2** was dissolved in THF to give a stock solution (2.7 × 10^−3^ mol L^−1^). Dye loading of the PS-NH_2_ was performed by addition of 200 μL of the dye solution to 5 mL of an aqueous suspension of the PS-NH_2_ (0.5 % w/v).

To determine the amount of dye incorporated into the PS nanoparticles, 300 μL of particle suspension were dissolved in 3 mL of THF followed by subsequent measurement of the absorption spectra of the transparent THF solutions. The average amount of dye incorporated into the nanomaterial was calculated from the absorbance measured at the dye's longest wavelength absorption maximum, using the Lambert-Beer law and the previously determined molar absorption coefficient of the dye in THF.

### NMR Spectroscopy

^1^H and ^13^C NMR spectra were recorded at 400 MHz and 100 MHz respectively using a Bruker Avance III 400 spectrometer (Bruker BioSpin GmbH, Rheinstetten, Germany) in DMSO-d_6_, referenced to the solvent for both proton and carbon spectra.

### Electron Microscopy

Transmission Electron Microscopy (TEM) images were obtained with a Hitachi 8100 electron microscope operating at 200 kV and 20 μA. Samples were dispersed in water and then a drop was placed on Formvar©/Carbon Coated Grid (200 mesh) and dried before examination. The dry nanoparticle size was estimated by measuring the average diameter of 100 nanoparticles by using ImageJ software.

### Optical Spectroscopy

Photophysical data were collected using optical spectroscopy apparatus previously described (Menezes et al., 2013; Crucho et al., 2020). Absorption and reflectance spectra were collected using a Shimadzu UV-3600 double beam spectrophotometer. Reflectance spectra were obtained for silica nanoparticles dispersed in BaSO4. Emission spectra were collected in a Jobin Yvon Fluorolog fluorescence spectrometer, respectively. Emission is independent of excitation wavelength. The luminescence temperature dependence measurements were acquired using a model liquid nitrogen cryostat (Janis Research). Fluorescence decays of dyes were measured by the single-photon timing method using nanoLED (IBH) excitation at 373 nm, with 500 ps pulse width. The electronic start pulses are shaped in a constant fraction discriminator (Canberra 2126) and directed to a time to amplitude converter (TAC, Canberra 2145). Emission wavelength (450 nm) is selected by a monochromator (Oriel 77,250) imaged in a fast photomultiplier (9814B Electron Tubes Inc.), the PM signal is shaped as before and delayed before entering the TAC as stop pulses. The analog TAC signals are digitized (ADC, ND582) and stored in a PC. For luminescent nanoparticles, fluorescence decays were measured by the single-photon timing method with laser excitation (365 nm) and emission at 550–620 nm. The setup consisted of a diode-pumped solid state (DPSS) continuous wave green Nd:YVO4laser (Millennia Xs, Spectra Physics) that pumped a mode locked Ti:sapphire laser (Tsunami, Spectra Physics, with tuning range 700–1,000 nm, output pulses of 100 fs, and 80 MHz repetition rate that can be reduced down to 4 MHz by a pulse picker) or mode locked DPSS Nd:YVO4green laser (Vanguard 2000-HM532,Spectra Physics) synchronously pumping two cavity dumped dye lasers (701, Coherent, delivering 3–4 ps pulses of about 40 nJ/pulse at 3.4 MHz) working with rhodamine 6G and DCM. Intensity decay measurements were made by an alternating collection of impulses and decays with the emission polarizer set at the magic angle position. Impulses were recorded slightly away from the excitation wavelength with a scattering suspension, thus defining the instrument response function (IRF). Particle samples were prepared either as suspensions in solvent (0.1–0.5% w/v) or immobilized in quartz plates using zeonex (20% in toluene) as binder. Temperature dependent time-resolved emission spectra were focused onto a spectrograph and detected on a sensitive gated iCCD camera (Stanford Computer Optics) with sub-nanosecond resolution. Solutions were prepared with concentrations in the 10^−5^-10^−4^ M range in different solvents, and samples were degassed using five freezepump-thaw cycles or bubbling Argon for 1 h. Films for optical characterization were prepared in zeonex or poly(vinylalcohol) matrix by drop-casting onto a quartz substrate with an emitter concentration of 1% (w/w). Prompt fluorescence quantum yields (Φ_PF_) were determined using the standard method for free dyes (vs. quinine sulfate in H_2_SO_4_ 0.01 M), and with the absolute method using an integrating sphere for luminescent nanomaterials. Delayed fluorescence quantum yields (Φ_DF_) using Φ_PF_ as internal reference and the ratio of the integrated areas of full luminescence decays acquired in degassed conditions, as given by Equation (1)

(1) $$\Phi_{\text{DF}}=\Phi_{\text{PF}}\left(\frac{{\text{I}}_{\text{DF}}}{{\text{I}}_{\text{PF}}}\right)$$

Phosphorescence, prompt fluorescence, and delayed emission (DF) spectra and decays were recorded using nanosecond gated luminescence and lifetime measurements (from 400 ps to 1 s) using either a high energy pulsed Nd:YAG laser emitting at 355 nm (EKSPLA) or a *N*_2_ laser emitting at 337 nm. Emission was focused onto a spectrograph and detected on a sensitive gated iCCD camera (Stanford Computer Optics) having subnanosecond resolution. PF/DF time resolved measurements were performed by exponentially increasing gate and delay times; details can be found elsewhere (Rothe and Monkman, 2003).

Dynamic Light Spectroscopy (DLS) was carried out on a Horiba nanoPartica SZ-100 V2 Nanoparticle Analyzer. Nanomaterials were suspended in water (1 mg/mL) and the analyzed samples were prepared by diluting the stock suspensions (1:100). Measurements were carried out at 90° scattering angle on quartz cuvettes at 25°C. All tests were run six times for 30 s and the average values were presented and particle size was calculated by fitting the correlation curves using solver mathematical software from the Stokes-Einstein equation.

### Live-Cell Imaging

MCF-7 cell line (European Collection of Authenticated Cell Cultures, ECACC) were cultured in DMEM (ThermoFisher Scientific) supplemented with 10% fetal bovine serum (FCS) (GIBCO) and 1% of penicillin- streptomycin (Sigma Chemical Co., St. Louis, MO) at the incubator with controlled temperature (37°C), humidity and CO2 levels (5%). For confocal and two-photon excitation microscopic studies, the cells were grown on Ibidi μ-Slide 8 well glass bottom with an initial density of 1 × 10^4^ cells per well. After cell confluency reached ~70%, 25–100 μg/mL of **PS3** and **PS4** or 1.5–200 μM of **2** were added separately in two different sets of experiments and maintained/cultured for a period of 24 h. After the desired incubation time, cells were carefully washed with DPBS (Thermo Fisher Scientific) and labeled with WGA-Alexa Fluor 633 according with instructions of the supplier (Thermo Fisher Scientific, Plasma Membrane and Nuclear Labeling dyes). A final washing step with DPBS was also included. The cells were imaged using a laser scanning confocal microscope (Leica TCS-SP5) equipped with a continuous Ar-ion, HeNe and a Ti:sapphire laser. WGA – Alexa Fluor 633 emission was collected from 640 to 700 nm and nanoparticle emission was collected from 500 to 600 nm. A 63 × (1.2 N.A.) water immersion objective was used in the experiments. Quantification analysis was carried out using the ImageJ software (version 1.48, http://imagej.nih.gov/ij/). For each image, individual cells are selected using membrane staining with WGA-Alexa 633, and a mask is attributed to each cell. The total fluorescence signal in the nanoparticle channel (500–600 nm) was integrated for cells incubated with and without (autofluorescence) nanoparticles, in order to determine the concentration dependent intracellular nanoparticle fluorescence signal and the fraction of cells that exhibit nanoparticle internalization. Number of cells analyzed per condition>40.

### Cytotoxicity Assay

The effect of the prepared nanomaterials on cell metabolic activity was determined by MTT [3-(4,5-dimethylthiazol-2-yl-2,5-tetrazolium bromide)] assay previously described (Raj and Das, 2017). Briefly, MCF-7 cells were plated at a density of 2 × 10^4^ cells per well into the 96-well plate and cultured for 24 h at 37°C in 5% CO_2_ atmosphere. Then, **PS3** and **PS4** at different concentrations (0–100 μg/mL) were added in duplicate and incubated for additional 24 h. After the incubation period, media was carefully replaced with 100 μL of fresh complete media without disturbing cell contents followed by addition of 20 μL of MTT solution (5 mg/mL) and incubated for 3–4 h. Finally, the formazan crystals formed in the wells were dissolved using 150 μL of MTT solvent and the absorbance was read at 590 nm using a microplate reader (bmg labtech 96 Spectrostar Nano).

## Data Availability Statement

All datasets generated for this study are included in the article/Supplementary Material.

## Author Contributions

Experimental: CC did the nanoparticle synthesis and characterization. JA did the photophysical measurements. AD did the organic dye synthesis. SP did the microscopy imaging and cytotoxicity studies. JB did cytotoxicity studies. PS performed cell culture preparation. Writing: The data was analyzed and validated by JA, FD, MB-S, and L-OP. The manuscript was written by JA and FD with contributions from all authors.

## Conflict of Interest

The authors declare that the research was conducted in the absence of any commercial or financial relationships that could be construed as a potential conflict of interest.

## References

[B1] Baldo M. O'Brien D. You Y. Shoustikov A. Sibley S. Thompson M. (1998). Highly efficient phosphorescent emission from organic electroluminescent devices. Nature 395, 151–154. 10.1038/25954

[B2] Baleizão C. Nagl S. Schaferling M. Berberan-Santos M. Wolfbeis O. (2008). Dual fluorescence sensor for trace oxygen and temperature with unmatched range and sensitivity. Anal. Chem. 80, 6449–6457. 10.1021/ac801034p 18651755

[B3] Behnke T. Würth C. Hoffmann K. Hübner M. Panne U. Resch-Genger U.. (2011). Encapsulation of hydrophobic dyes in polystyrene micro and nanoparticles via swelling procedures. J. Fluoresc. 21, 937–944. 10.1007/s10895-010-0632-2 20213240

[B4] Bui T. Goubard F. Ibrahum-Ouali M. Gigmes D. Dumur F. (2018). Recent advances on organic blue thermally activated delayed fluorescence (TADF) emitters for organic light-emitting diodes (OLEDs). Beilstein J. Org. Chem. 14, 282–308. 10.3762/bjoc.14.18 29507635 PMC5815274

[B5] Chen W. Song F. (2019). Thermally activated delayed fluorescence molecules and their new applications aside from OLEDs. Chin. Chem. Lett. 30, 1717–1730. 10.1016/j.cclet.2019.08.032

[B6] Crucho C. Avó J. Nobuyasu R. Pinto S. Fernandes F. Lima J.. (2020). Silica nanoparticles with thermally activated delayed fluorescence for live cell imaging. Mater. Sci. Eng. C 109:110528. 10.1016/j.msec.2019.110528 32228970

[B7] Dias F. Penfold T. Monkman A. (2017). Photophysics of thermally activated delayed fluorescence molecules. Methods Appl. Fluoresc. 5:012001. 10.1088/2050-6120/aa537e 28276340

[B8] Dias F. Santos J. Graves D. Data P. Nobuyasu R. Fox M.. (2016). The role of local triplet excited states and D-A relative orientation in thermally activated delayed fluorescence: photophysics and devices. Adv. Sci. 3:1600080. 10.1002/advs.201600080 27981000 PMC5157178

[B9] Dias F. B. Bourdakos K. N. Jankus V. Moss K. C. Kamtekar K. T. Bhalla V.. (2013). Triplet harvesting with 100% efficiency by way of thermally activated delayed fluorescence in charge transfer OLED emitters. Adv. Mater. 25, 3707–3714. 10.1002/adma.201300753 23703877

[B10] Lee J. Gomez I. Sitterle V. Meredith J. (2011). Dye-labeled polystyrene latex microspheres prepared via a combined swelling-diffusion technique. J. Coll. Interf. Sci. 363, 137–144. 10.1016/j.jcis.2011.07.047 21839463

[B11] Lee J. White A. Rice D. Smith D. (2013). *In vivo* imaging using polymeric nanoparticles stained with near-infrared chemiluminescent and fluorescent squaraine catenane endoperoxide. Chem. Commun. 49, 3016–3018. 10.1039/c3cc40630j 23467338 PMC3633569

[B12] Li L. Wang W. Tang J. Wang Y. Liu J. Huang L.. (2019). Classification, synthesis, and application of luminescent silica nanoparticles: a review. Nanoscale Res. Lett. 14:190. 10.1186/s11671-019-3006-y 31165269 PMC6548908

[B13] Li T. Yang D. Zhai L. Wang S. Zhao B. Fu N.. (2017). Thermally activated delayed fluorescence organic dots (TADF Odots) for time-resolved and confocal fluorescence imaging in living cells and *in vivo*. Adv. Sci. 4:1600166. 10.1002/advs.201600166 28435770 PMC5396166

[B14] Luo X. Meng J. Li B. Peng A. Tian Z. (2019). Development of fluorescent nanoparticles with aggregation-induced delayed fluorescence features, improved brightness and photostability for living cells imaging. New J. Chem. 43, 10735–10743. 10.1039/C9NJ01945F

[B15] Mader H. Wolfbeis O. (2010). Optical ammonia sensor based on upconverting luminescent nanoparticles. Anal. Chem. 82, 5002–5004. 10.1021/ac1007283 20481605

[B16] Menezes F. Fedorov A. Baleizão C. Valeur B. Berberan-Santos M. (2013). Methods for the analysis of complex fluorescence decays: sum of Becquerel functions versus sum of exponentials. Methods Appl. Fluoresc. 1:015002. 10.1088/2050-6120/1/1/015002 29148435

[B17] Montalti M. Prodi L. Rampazzo E. Zaccheroni N. (2014). Dye-doped silica nanoparticles as luminescent organized systems for nanomedicine. Chem. Soc. Rev. 43, 4243–4268. 10.1039/C3CS60433K 24643354

[B18] Ni F. Zhu Z. Tong X. Xie M. Zhao Q. Zhong C.. (2018). Organic emitter integrating aggregation-induced delayed fluorescence and room-temperature phosphorescence characteristics, and its application in time-resolved luminescence imaging. Chem. Sci. 9, 6150–6155. 10.1039/C8SC01485J 30090303 PMC6053954

[B19] Ni F. Zhu Z. Tong X. Zeng W. An K. Wei D.. (2019). Hydrophilic, red-emitting, and thermally activated delayed fluorescence emitter for time-resolved luminescence imaging by mitochondrion-induced aggregation in living cells. Adv. Sci. 6:1801729. 10.1002/advs.201801729 30886801 PMC6402405

[B20] Nobuyasu R. Ward J. Gibson J. Laidlaw B. Ren Z. Data P. (2019). The influence of molecular geometry on the efficiency of thermally activated delayed fluorescence. J. Mater. Chem. C 7, 6672–6684. 10.1039/c9tc00720b

[B21] Peng J. Li J. Xu W. Wang L. Su D. Teoh C.. (2018). Silica nanoparticle-enhanced fluorescent sensor array for heavy metal ions detection in colloid solution. Anal. Chem. 90, 1628–1634. 10.1021/acs.analchem.7b02883 29275622

[B22] Raj R. Das S. (2017). Development and application of anticancer fluorescent CdS nanoparticles enriched *Lactobacillus* bacteria as therapeutic microbots for human breast carcinoma. Appl. Microbiol. Biotechnol. 101, 5439–5451. 10.1007/s00253-017-8298-1 28455616

[B23] Rothe C. Monkman A. (2003). Triplet exciton migration in a conjugated polyfluorene. Phys. Rev. B 68:075208 10.1103/PhysRevB.68.075208

[B24] Saei A. Yazdani M. Lohse S. Bakhtiary Z. Serpooshan V. Ghavami M. (2017). Nanoparticle surface functionality dictates cellular and systemic toxicity. Chem. Mater. 29, 6578–6595. 10.1021/acs.chemmater.7b01979

[B25] Tsuchiya Y. Ikesue K. Nakanotani H. Adachi C. (2019). Photostable and highly emissive glassy organic dots exhibiting thermally activated delayed fluorescence. Chem. Commun. 55, 5215–5218. 10.1039/C9CC01420A 30896723

[B26] Uoyama H. Goushi K. Shizu K. Nomura H. Adachi C. (2012). Highly efficient organic light-emitting diodes from delayed fluorescence. Nature 492, 234–238. 10.1038/nature11687 23235877

[B27] Wang H. Xie L. Peng Q. Meng L. Wang Y. Yi Y.. (2014). Novel thermally activated delayed fluorescence materials–thioxanthone derivatives and their applications for highly effi cient OLEDs. Adv. Mater. 26, 5198–5204. 10.1002/adma.201401393 24903266

[B28] Wang X. Achatz D. Hupf C. Sperber M. Wegener J. Bange S. (2013). Imaging of cellular oxygen via two-photon excitation of fluorescentsensor nanoparticles. Sensor. Actuat. B Chem. 188, 257–262. 10.1016/j.snb.2013.06.087

[B29] Wolfbeis O. (2015). An overview of nanoparticles commonly used in fluorescent bioimaging, Chem. Soc. Rev. 44, 4743–4768. 10.1039/c4cs00392f 25620543

[B30] Xiong X. Song F. Wang J. Zhang Y. Xue Y. Sun N.. (2014). Thermally activated delayed fluorescence of fluorescein derivative for time-resolved and confocal fluorescence imaging. J. Am. Chem. Soc. 136, 9590–9597. 10.1021/ja502292p 24936960

[B31] Yang Z. Mao Z. Xie Z. Zhang Y. Liu S. Zhao J.. (2017). Recent advances in organic thermally activated delayed fluorescence materials. Chem. Soc. Rev. 46, 915–1016. 10.1039/c6cs00368k 28117864

[B32] Yu J. Rong Y. Kuo C. Zhou X. Chiu D. (2017). Recent advances in the development of highly luminescent semiconducting polymer dots and nanoparticles for biological imaging and medicine. Anal. Chem. 89, 42–56. 10.1021/acs.ana.lchem.6b04672 28105818 PMC5682631

[B33] Zhang Y. Zhuang X. Gu X. Zhao J. (2015). Synthesis of polyacrylonitrile nanoparticles at high monomer concentrations by AIBN-initiated semi-continuous emulsion polymerization method. Eur. Polym. J. 67, 57–65. 10.1016/j.eurpolymj.2015.03.057

[B34] Zhao Q. Huang C. Li F. (2011). Phosphorescent heavy-metal complexes for bioimaging. Chem. Soc. Rev. 40, 2508–2524. 10.1039/C0CS00114G 21253643

[B35] Zhu Z. Tian D. Gao P. Wang K. Li Y. Shu Z.. (2018). Cell-penetrating peptides transport noncovalently linked thermally activated delayed fluorescence nanoparticles for time-resolved luminescence imaging. J. Am. Chem. Soc. 140, 17484–17491. 10.1021/jacs.8b08438 30525541

