# Supplementary material for: TADF Dye-Loaded Nanoparticles for Fluorescence Live-Cell Imaging
Source: Front Chem. 2020 May 8;8:404. doi: 10.3389/fchem.2020.00404 (PMC7227253; doi:10.3389/fchem.2020.00404)
Supplement: Supplementary file 1 [file Table_1.DOCX]

**Supplementary Material**

TADF Dye-loaded Nanoparticles for Fluorescence Live-cell Imaging

Carina I.C. Crucho,^1^ João Avó,^1^* Ana Diniz,^1^ Sandra N. Pinto,^1^ José Barbosa,^1^ Poppy O. Smith,^2^ Mário Nuno Berberan-Santos,^1^ Lars-Olof Pålsson,^2^ Fernando B. Dias^3^*

^1^IBB-Institute for Bioengineering and Biosciences, Instituto Superior Técnico, Universidade de Lisboa, Portugal

^2^Department of Chemistry, Durham University, South Road, Durham, DH1 3LE, United Kingdom

^3^Department of Physics, Durham University, South Road, Durham, DH1 3LE, United Kingdom

Contents

[NMR spectroscopy 2](#_Toc33092328)

[TEM images of PS nanoparticles 3](#_Toc33092329)

[DLS spectroscopy 4](#_Toc33092330)

[Optical spectroscopy 5](#_Toc33092331)

[Live-cell studies 7](#_Toc33092332)

# NMR spectroscopy


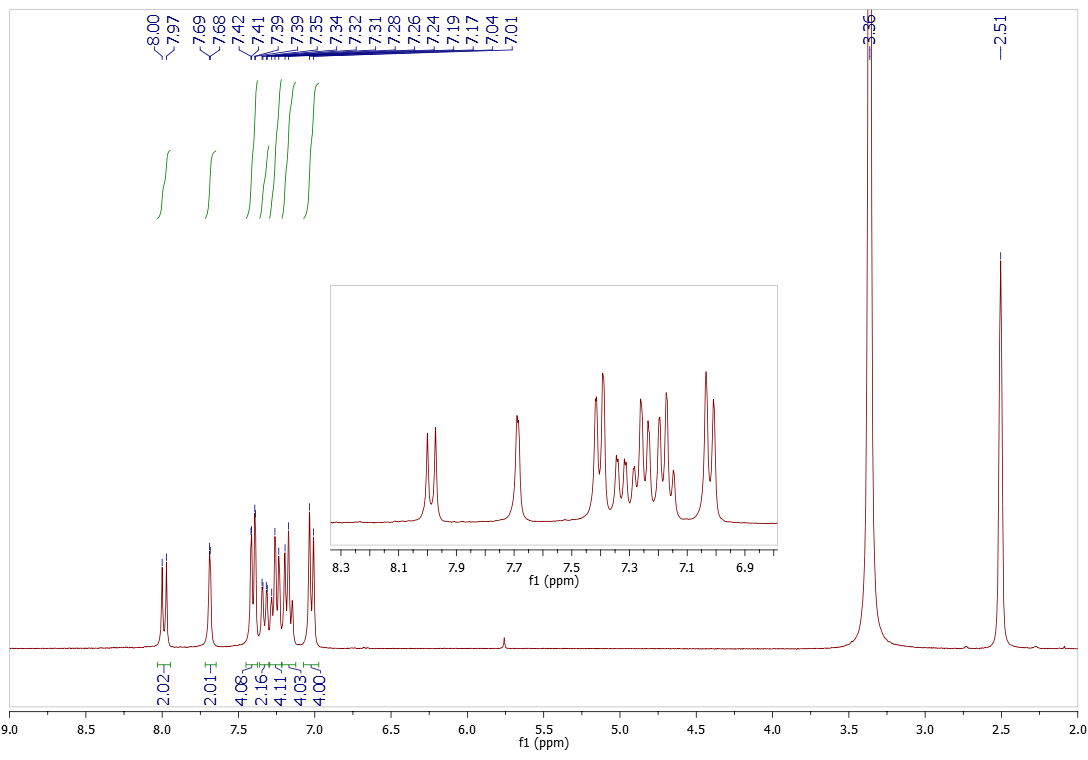


**Figure S1** - ^1^H NMR spectrum of dye **1** in DMSO-d_6_.

# TEM images of PS nanoparticles

**Figure S2** – TEM images of nanoparticles loaded with dye **1**: PS1 (a) and PS2 (b). Scale bar = 100 nm.

**Figure S3** – TEM images of nanoparticles loaded with dye **2**: PS3 (a) and PS4 (b). Scale bar = 100 nm.

DLS spectroscopy

**Figure S4** – Size distribution and correlation curves (insets) obtained by dynamic light scattering spectroscopy in water for dye loaded nanoparticles: **PS1** (a); **PS2** (b); **PS3** (c) and **PS4** (d).

Evident from the DLS data depicted in Fig. S4, neutral PS nanoparticles do not form stable suspensions in aqueous media, probably due to their highly hydrophobic surface. Instead, it is shown that a large portion of those particles are aggregated in small clusters, as demonstrated by the two-population distribution (Fig. S4a and S4c).

# Optical spectroscopy

**Figure S5** – Steady-state emission spectra of dye **1** (a) and dye **2** (b) collected in different organic solvents and water. Insets show normalized spectra.

**Table S1** - Prompt fluorescence and total quantum yield (ϕ_PF_, ϕ_PL_), prompt and delayed emission lifetime (τ_PF_, τ_DF_) and wavelength (λ_em_) of dyes **1** and **2**, and luminescent nanomaterials **PS1-4**, measured in aqueous media

|  | Solvent/Film | λ_em_ (nm) | Φ_PF_ | Φ_PLQY_ |
| --- | --- | --- | --- | --- |
| Dye **1** | Toluene  H_2_O  Polystyrene | 580  563  545 | 0.05  0.02  0.05 | 0.15  n.d.  0.14 |
| Dye **2** | Toluene  H_2_O  Polystyrene | 569  629  552 | 0.04  <0.01  0.20 | 0.05  <0.01  0.32 |

**Figure S6** – Absorption spectra of dissolved dyes and dye-loaded nanoparticles in THF/water 9:1 mixtures; a) dye **1**, **PS1** and **PS2**, [**1**] = 2.8 × 10^-5^ M, [**PS1**] = 0.13 mg/mL, [**PS2**] = 0.2 mg/mL; b) dye **2**, **PS3** and **PS4**, [**2**] = 5.0 × 10^-5^ M, [**PS3**] = 0.12 mg/mL, [**PS4**] = 0.2 mg/mL.

**Figure S7** – a) Luminescence decay of **PS4** dispersed in poly(vinylalcohol) film, collected as a function of temperature; b) Variation of time-resolved emission intensity as a function of excitation intensity.

# Live-cell studies


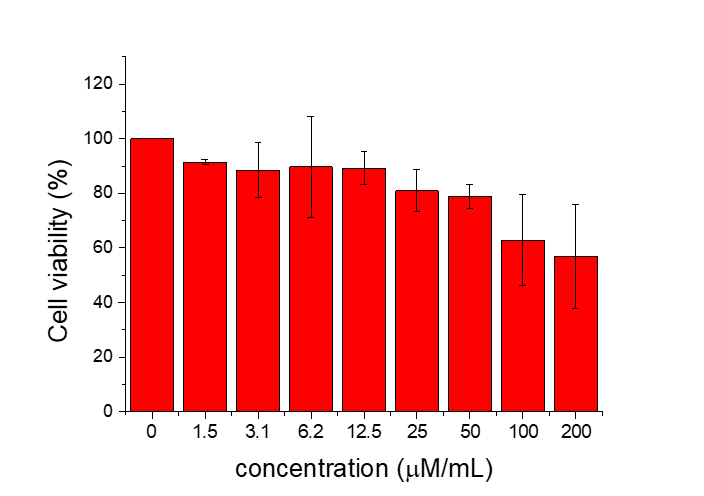


**Figure S8** - Effect of dye **2** on MCF-7 cell viability. Cells were treated with various concentrations of **2** (0-200 μM). The viability was measured by the MTT assay. The percentages refer to relative cell viability represented as percentage of control. Incubation time = 24 hours.

**Figure S9** - Confocal microscopy images of MCF-7 cells incubated for 24h in the presence of **PS3** (a) and **PS4** (b) at a concentration of 100 µg/mL in incubation medium.

**Figure S10** - Confocal microscopy images of MCF-7 cells incubated for 24h in the presence of **PS3** (a) and **PS4** (b) at a concentration of 50 µg/mL in incubation medium.

**Figure S11** - Confocal microscopy images of MCF-7 cells incubated for 24h in the presence of **PS3** (a) and **PS4** (b) at a concentration of 25 µg/mL in incubation medium.

**Figure S12** - Average intracellular nanoparticle fluorescence, measured as a function of concentration of **PS3** (red) and **PS4** (blue) in the incubation medium. Minimum cell count = 50.
